# Fever—Its Seat and Nature

**Published:** 1826-07

**Authors:** 


### MEDICO-CHIRURGICAL

### REVIEW.

VOL. IX.] Analytical Series. [No. 25.

" Nec tibi quid liceat sed quid fecisse decebit

" Occurrat, mentemque domat respectus honesti."

VOL. V.] JULY 1, 1826. [No. 9.

[NEW SERIES.]

### I.

1. *Conversations on the Theory and Practice of Physiological Medicine; or, Dialogues between a Savant and a young Physician, a Disciple of Professor Broussais; containing a Concise Exposition of the New Medical Doctrine, &c. &c.* Translated from the French. 8vo. pp. 326. December, 1825.

2. *An Inquiry into the Seat and Nature of Fever; as deducible from the Phenomena, Causes, and Consequences of the Disease, the Effects of Remedies, and the Appearances on Dissection.* By HENRY CLUTTERBUCK, M.D. &c. &c. *Second Edition.* 8vo. pp. 494. 1825.

3. *Examen des Doctrines Médicales, et des Systémes de Nosologie.* Par F. S. V. BROUSSAIS, M.D. &c. &c. Two volumes, 8vo. Paris, 1821.

IT is now some 20 years since, on the demise of honest Brown and his doctrines, it was universally acknowledged that there was a vacancy for a new theory of fever. On this occasion two candidates came forward—Dr. Clutterbuck in this country—and Dr. Broussais in France. Our fellow citizen had the start, and the *brain* being, as he thought, unoccupied, he placed the seat of fever there at once. Broussais was obliged to take up an humbler station—and he pitched upon the stomach and bowels, as the theatre for his future operations and speculations. Other candidates, but, *minoris notæ*, have since endeavoured to establish the nature and fix the seat of fever, with indifferent success. Some have selected one organ, and others another, for the throne of this mysterious disease—and some (as Marcus and Mills) have given fever a kind of roving commission, or *passe partout*, to establish its head-quarters wherever it may find the friendliest reception among the viscera of the body.—The two great leaders at the head of our list, have each a considerable number of disciples and proselytes—but those of Broussais are infinitely more numerous, noisy, and intolerant than the followers of Clutterbuck in this country. They are as tenacious and blood-thirsty as the swarms of leeches which they employ—and woe to the unfortunate *entologist* who ventures to dispute the omniscience of their oracle! But both parties are intolerant—and strange to say, they are more inveterate against the *eclectics*, or those who endeavour to select the truth from among the errors of the adverse systems, than they are against their direct opponents. Broussais anathematises the *eclectic* system as "the anarchy of science"—"because it furnishes a complete proof of the imperfection of medical doctrines;"—while our worthy countryman attributes the vague and vacillating treatment of fever to the non-adoption of the fixed principle laid down by him in his theory of the disease. Now, this is very hard upon the *eclectics*; for it must be admitted, even by the rival candidates for supremacy themselves, that both systems cannot be right—and, therefore, the probability is, that truth lies between—and that perfection is the lot of neither. The seat of fever, in fine, is like the rainbow—or the horizon, which

> "Allures from far, but as we follow flies."

We are ready to grant, however, that the writings and investigations of Dr. Clutterbuck, Dr. Broussais, and all those who have endeavoured to deprive fever of its *idiopathic* title, and reduce it to the character of a symptomatic affection, have done a vast deal of good, by drawing the attention of pathologists and practitioners to those *local affections* which, if they do not *cause*, very often *accompany* the fever, and impress on it the chief modifications by which our indications of treatment are to be guided. On this account we deem it desirable that a more comprehensive knowledge of the facts and arguments brought forward by the two leaders, should be diffused generally through the profession, in a form that may secure an attentive perusal. We do not speak unguardedly when we aver that, not one in fifty of the profession in this country has a clear idea of the doctrine of the French pathologist—and that even the doctrine of Clutterbuck is far from being generally understood among our practitioners—only one small edition of his work having been distributed during a period of nearly 20 years. The succinct *exposé* which we shall attempt of these two systems, will, we hope, prove a vehicle for the communication and concentration of a vast mass of important information, which, being scattered through many volumes, is inaccessible to, or unavailable by, the great body of medical practitioners. We shall endeavour to exhibit our author's line of argument and the spirit of his doctrine in the text, reserving the notes for our comments, when deemed necessary.

Dr. Clutterbuck's general or preliminary observations need not detain us long. They contain truths admitted by all. Thus he maintains that inflammation (a disorder of the vascular system chiefly) is the immediate cause of nine in ten of the great fatal maladies which afflict mankind. The following passage is substantially correct in the principle therein maintained; but, as will be seen in the sequel, Dr. Clutterbuck, as well as M. Broussais, has made it the pillar on which, almost, the whole weight of his edifice rests.

> " A disease may consist merely in *disordered action,* occasioning either altered feelings, or disturbance of functions, or both; without any perceptible *alteration of structure* in the part affected. In such cases, dissection after death can afford us no insight into either the *seat* or *nature* of the disease." 3.

"Inflammation," says Dr. C., "is *primarily* and essentially a state of *disordered action merely*, though leading eventually to *alteration of structure*"* We agree with Dr. C. that changes may take place at, or subsequent to, death, which sometimes lead astray—taking away, in some cases, the marks of *very recent* inflammation—and producing appearances, in other cases, that seem the effects of inflammation, where none really existed. Farther we agree, to a certain extent, with our author, that, "dissection, for the most part, shews the *consequences* only of diseased action, and gives but little insight into the nature of the action upon which those consequences depend."†

* Is it a fact, that we characterise by the term *inflammation* that which presents no visible alteration from the healthy state? Let us take the conjunctiva, for example;—do we say it is *inflamed* when nothing unusual can be seen in it?—Is there no *alteration* of structure when a tissue is changed from transparency to opacity—from whiteness to blood-redness? Surely every part that is *inflamed* (according to any definition which Dr. C. may select) is *altered* in its structure from a state of health. We grant, indeed, that *disordered action* may, and does, precede this visible change of structure; but it is not inflammation till something can be seen.

† We admit, and have often urged this point, that the appearances on

Morbid anatomy, therefore, in Dr. C's. opinion, "is of limited use and application in the practice of medicine." Diseases must be judged of chiefly by their symptoms. But diseased actions are not always accompanied by *sensation*. The lungs, brain, and other parts have been found disorganized after death, where no pain was previously complained of. If diseases cannot always be detected by feelings of the organ, they are sometimes discoverable by the affection of other parts that are sympathetically connected with them. "Thus, we are enabled to trace many affections of the stomach, of the vascular system, and of other remote parts, to a disordered state of the brain." 7.* Dr. C. goes on to observe that no part can undergo any material alteration, without some change in its functions. Thus, if the *brain* be the organ affected, sensation, voluntary motion, and vital energy (its functions among others) will suffer in proportion to the extent and degree of topical disease. This leads our author into many important remarks on primary and symptomatic affections and their symptoms, for which we must refer to the work itself. A division of diseases, he observes, has been made into local and general, and in the latter class, *idiopathic* fever is placed—the only reason for which is, in his opinion, "the more early and general disturbance that takes place in this, in comparison with other diseases." This early general disturbance, he thinks, may be accounted for on *local* princi-

---

dissection are more frequently the *consequences* than the *causes* of the disease of which the patient dies. But surely the morbid appearances very often give us an insight into the nature of the actions that were going on during life, and which led to the production of these *post mortem* phenomena. Look at the membranes of the brain—at the pleura—the peritoneum—the parenchymatous structure of various organs—do we not every day recognize in them the undoubted proofs of previous inflammation—although we cannot agree as to the precise *nature* of inflammation itself?

* We will do Messrs. Clutterbuck and Broussais the justice to say, that they have not only fortified themselves with much generalship, but they have provided themselves with most convenient postern gates for escape, in the event of being stormed in their respective garrisons. Thus, if Dr. Clutterbuck comes to a man in fever, with constant vomiting, pain and tenderness in the epigastrium, tongue like a beef-steak, but with the intellectual faculties perfectly clear, he tells us—" this is manifestly only a *symptomatic* affection of the stomach, the source of the phenomena being in the brain, as I have demonstrated in my work." On the other hand, if Professor Broussais visits a patient in the next bed, with raging delirium, black tongue, ferretty eyes, &c. but without a single symptom of gastric affection, he exclaims, "Ah! ma fois, here is a disorder of the sensorial functions purely *symptomatic* of gastro-enteritis, which is the fons et origo of all the phenomena which now excite your attention." Each leader supports his position with a host of arguments and analogies, and the poor eclectic is left in the dark, to struggle out of the labyrinth as he can!

ples. The simplicity or the complexity of an organ affected makes great difference in the phenomena of its diseases. Thus, if the pleura merely be inflamed, the symptoms are few—" and consist in little more than pain in the affected part, with an excited state of general vascular action." "In peripneumony, a much more extensive disorder takes place in the system, from the great importance of respiration in the general economy." "In this point of view, no organ will bear comparison with the brain, which exerts a paramount influence over all.*" Hence, Dr. C. observes, it is to be expected that "when the general substance of the brain is suffering from disease, the whole system will be drawn into consent, and a general disturbance of functions ensue."

CHAP. I. *Seat of Fever.* In endeavouring to assign the *primary* seat of fever, Dr. C. finds it necessary to examine the phenomena of the disease—leaving out of the question, for the present at least, those fevers which are evidently symptomatic of local inflammation, as of pneumonia, &c. In *idiopathic fever*, so called, " many of the most important functions are observed to be *disturbed.*" The functions of digestion and assimilation are nearly annihilated, while that of absorption is increased. In many instances, the functions of the vascular

---

* Dr. Clutterbuck takes care to seize on every analogy, however remote, to strengthen his doctrine. Many of these analogies are more specious than solid—and some of them are absolutely erroneous. Thus, simplicity of structure does not always secure simplicity or paucity of morbid phenomena, as our author would wish us to believe. The pleura itself, when inflamed, produces more general distress and excitement through the system, than when the parenchymatous structure of the lung is the seat of disease. So inflammation of the peritoneum, which is a simple serous tissue, is a much more dangerous disease than inflammation of the parenchymatous structure of the liver or spleen—or of the mucous membrane, which is a far more complicated structure. The serous membranes, which are the simplest structures, have many more sympathetic connexions than the glandular or parenchymatous organs themselves. Peritoneal inflammation will completely suspend the function of the organ over which it runs—even of the mucous membrane beneath, as we see in enteritis. If we ascend to the head, these observations are still more strongly exemplified. The intellectual functions, as well as the corporeal, are infinitely more disturbed by inflammation of the membranes—especially the arachnoid membrane of the brain, than by inflammation of the substance—if, indeed, the substance be susceptible of inflammation, which is much doubted by some pathologists. Thus we see how badly these analogies and arguments of our author, will stand the test of rigid examination, although with the student, and with the inattentive or inexperienced reader, they may pass for strong auxiliaries to the doctrine advocated. It will be our business, in this article, to detect fallacies and expose sophistry, wherever we find them—nor will our labour be useless when so employed.

system, and of the kidneys, liver, and intestinal canal, continue to be performed "nearly as in health"—at other times, one or more of them are greatly disordered. But although the phenomena of fever appear, at first view, to be various and complicated, Dr. C. thinks that a careful examination of them will enable us to distinguish the *primary and essential* symptoms from those which are only secondary occasional occurrences.

Dr. Clutterbuck, in order to avoid any suspicion of colouring the phenomena to suit his theory, takes for his text, the concise but faithful portrait drawn by Fordyce. This extract is necessary for our purpose also, and we shall give it as Dr. C. has printed it, the words in italics being meant by him to shew the true *pathognomic* characters of fever.

" *First Stage.* 'The attack of fever, whenever it can be distinctly observed, is announced by the following symptoms, in greater or less degree.

"' *(a) Languor,* weariness, weakness, *insensibility of the extremities; blindness* and *insensibility* in the other organs of sensation; cold and trembling; *pain in the back.*

" ' *(b) Horripilatio;* the skin pale, dry, and of a dusky colour; a dry, foul tongue, and thirst; transparent urine; costiveness, and suppression of other secretions; paleness and dryness in ulcers; a small obstructed pulse, sometimes intermitting; pain in the limbs, joints, and *forehead; delirium.*

" ' *(c) Anxiety;* oppression and swelling about the præcordia; frequency of the pulse; quick and laborious respiration, sometimes with a cough; rigor, and horror; thirst, flatulency, loss of appetite, *nausea,* and *vomiting.*'

" These are the symptoms which constitute the first stage of fever, and are properly characteristic of it: 'according to the violence of these symptoms at any time of the disease, the fever is violent; and when they are entirely carried off, it is cured.'

" *Second Stage.* The symptoms of the *first stage,* above enumerated, soon give place to those of the *second stage,* which succeed each other in the following order:—'Rigor and horror; heat arising from the præcordia, and diffused over the body irregularly, unequally, and with flushing; a strong, full, *obstructed* pulse; or a very frequent, small one; *pain in the head* and joints; *stupor* and *delirium; universal soreness;* redness arising in different parts irregularly; the urine high coloured, but transparent; sweating in the head and breast, or over the whole body; partial secretions.'

" *Third Stage.* 'At last, the pulse becomes free; all the secretory organs are relaxed: hence the skin grows soft and moist, and returns to its natural colour; the tongue likewise is soft and moist, the belly is open, and the urine in greater quantity; if transparent when discharged, after a little time it becomes turbid and opaque, and at last deposits a copious sediment; the secretions are often greatly increased; there arises a copious and universal sweat, or a purging, or a great flow of urine.'

"The frequency of the pulse, and all the other symptoms of the first and second stage, gradually subsiding, the patient recovers his health, but is considerably weakened."

We need not notice the extracts which Dr. C. has taken from various other authors, corroborating the above description, since we fully admit its general correctness, and shall join issue with Dr. C. on the document as it stands.

Dr. Clutterbuck's doctrine is then as follows:

"1. *Idiopathic* fever, as it is termed, is essentially a *local* disease, and not *primarily* a general affection of the system, as has commonly been believed.

"2. The proper and exclusive seat of it, is the brain.

"3. It consists in actual inflammation of the general cerebral *substance.*" 39.

Now, in this place we must take leave to observe, that when Dr. Clutterbuck brought forward his doctrine in the first edition of his work, he maintained that fever was inflammation of the brain *or its membranes*—agreeing most cordially with Cullen, that all idea of a distinction between inflammation of the *substance* and of the *membranes* of the brain was futile.* But now it is quite another story. Fever consists of inflammation of the *substance* of the brain—and the distinction between the membranous and parenchymatous inflammations, not only "really exists," but is "discoverable by the symptoms."—*2d Ed. p.* 169. The reader may be curious to know the reason of this discrepancy of doctrine in the two editions. To us it is very obvious. Twenty years ago, the pathological anatomy of the brain had not arrived at its present pitch of minuteness. Since that period, the effects of inflammation on the *membranes* of the brain have been as clearly demonstrated as the effects of the same disease on any other structure of the body.† And as *post-mortem* examinations of fever patients have not shewn these effects, the *membranes* have been discarded, and we are led into the *substance* of the brain, which, being impermeable by the finest injections, is not accountable for what passes there in fever—is not amenable to the scalpel, —and is, therefore, the very best theatre possible for placing a proximate cause whose existence is to be proved by *reasoning*, and not by the evidence of the senses.

---

* See page 140-1 of First Edition.

† See our Analysis of Duchatelet, for instance, on Arachnitis, Vol. 2, p. 195, et seq. last Series.

Here, however, Dr. Clutterbuck's memory has been rather defective, and we must refresh it. We know that the effects of inflammation on the *substance* of the brain are not so numerous or so easily ascertained as in the *membranes;* but still they have been ascertained by unbiassed pathologists, and they do not correspond in symptoms with fever.* In Dr. Clutterbuck's anxiety to draw the distinction between parenchymatous and membranous inflammation of the brain (which he formerly denied) he has been led, not only into error, but into inconsistency. Thus, at p. 169 of the present edition, he remarks:—" An affection of the membranes *exclusively*, can have no power to produce disturbance in the *functions* of the brain." And yet he almost immediately afterwards observes—" undoubtedly inflammation of membranes may, by contiguity of parts, and continuity of vessels, be a source of irritation to the brain, and thus, in some degree, *disorder its functions*, without the existence of actual inflammation." Surely these two passages are in direct contradiction to each other. The truth of the first sentence we dispute. Every pathologist who has watched the living phenomena and compared them with the *post mortem* appearances, well knows that inflammation or even irritation of the serous membranes, especially of the brain, will disturb, and that most powerfully, the functions of the encephalon, both physical and intellectual. Our readers will see, by turning to our account of Parent Duchatelet's work on Meningitis, that, in inflammation of the membranes, especially of those covering the hemispheres, *delirium*, and every lesion which Dr. C. attributes to inflammation of the substance of the brain, are " early and regular characteristic phenomena." In short, the whole of the investigations carried on by our neighbours as well as ourselves, during the last fifteen years, contradict, most completely, the following assertion of Dr. Clutterbuck's. " Simple inflammation of the *membranes* of the brain does *not*, therefore, produce phrenitis, which, in the ordinary acceptation of the term, is characterized by great disturbance, in one, at least, of the sensorial functions." —171. Dr. C. may characterize *phrenitis* as he pleases, but we maintain that inflammation of the *membranes* will produce *delirium* and great disturbance, not of one only, but of almost all the sensorial functions. For proofs, we refer to histories and dissections—whereas Dr. C. gives us nothing but argument and assertion.

---

† See our review of Lallemand's work on the Brain, for instance, vol. iii. p. 465, et seq. last series. See also the writings of Roston and many other able pathologists.

Dr. Clutterbuck has taken up a great portion of his volume in describing the disturbed state of the sensorial functions in fever, as those of sense, voluntary motion, and intellect. No one can deny—no one has ever denied, or indeed failed to notice, these lesions of function in fever—but the question at issue is, "are they owing to *inflammation* of the brain?"—This we deny. At the same time we are ready to admit, and, indeed, we have always maintained, that the *sentient system* is that which, from observation of the phenomena, appears to be the first implicated in fever—or the first on which the cause of the disease operates. This circumstance, and the universality of influence which the nervous system exerts on all the functions and structures of the body; have given Dr. Clutterbuck's theory a decided superiority over that of Broussais, and render it much more capable of defence, and, consequently, much more difficult of demolition. Nevertheless we shall proceed to the trial.

First, then, we propose to shew that fever does not present the phenomena (in the living or the dead body) of inflammation of the brain, which we shall term, for shortness, phrenitis; and, secondly that, although phrenitis exhibits the general phenomena of fever, *at certain periods or stages of fever*,—yet, that it does not correspond with fever, in its origin, progress, termination, cure, or *post mortem* appearances.

The first dawning of idiopathic fever, as it steals on, almost imperceptibly, offers convincing proof that the brain and nervous system are affected by something that *depresses* their natural energy, and *weakens* the functions depending on them. The individual complains of languor and debility of mind and body—his limbs totter—the tongue trembles—the sight wavers—the senses are obtunded, depraved, or sometimes lost—the pulse is weak—the breathing quick, feeble, and interrupted by sighing—the features shrunk—the eyes lack lustre—the secretions diminished—the appetite gone—the urine limpid and small in quantity—the skin cool, pale, dry—the bowels costive. Can any train of symptoms be more indicative of a *depression* of the sensorial functions—and of all the other functions connected with the sensorial? This state will continue for days—nay, for weeks, with very little variation. The scene then gradually changes, until what is called a *reaction* is established, in which *every* one of the former symptoms is *reversed*. We say *every* one, not even excluding the intellectual functions. For, although we do not maintain that the mind, from a state of depression, rises into that of preternatural vigour, yet it certainly changes into a state the reverse of the former, inasmuch as it becomes morbidly *irritable*, and all the senses morbidly acute. This state of reaction goes on, with evening exacerbations and morning remissions—or with complete intermissions on alternate days, for weeks together—gradually going off, without the least aid from medicine, and leaving the patient almost a skeleton, requiring some months for convalescence and ultimate recovery.

This is the *general portrait* of idiopathic fever—and it is by this that we are to be guided—not by anomalous cases or exceptions, where inflammation of this or that organ may have taken place at an earlier or later period of the fever. If we draw our conclusions from this last class, we shall readily confound right and wrong.

How does the above portrait correspond with the fever produced by local inflammation, with which it is confounded by Clutterbuck, Broussais, and many other physicians? We say it does not correspond with these symptomatic fevers. In these last, there is an evident local inflammation, as of the lungs, liver, stomach, &c. evinced by the proper symptoms of local pains—and of a disproportioned derangement of function in the organ affected. It is very true that an inflammation of the bowels—nay, a compound fracture of the leg, may produce a fever which, at a certain *advanced stage*, will present all the phenomena of idiopathic fever, at a *similar stage*; but look at the origin, course, and cause of each, and, while the resemblance is admitted, their identity will be denied. Look at the treatment required. In idiopathic fever, we may remain passive spectators, and five out of six will recover. In phrenitis, or other inflammation of an internal organ, if we look passively on, five out of six will die!

It is true that when the brain (the source of sensation, motion, and intellectual operations) is inflamed, we have then a disease which, though local in its seat, is general in its symptoms—and it is this circumstance that has given all the plausibility to the theory, against which we are contending. But still, if we examine minutely the fever produced by inflammation of the brain, we shall find that it resembles, essentially, all the other symptomatic fevers, except in the universality of its symptoms, and that it does *not* correspond with the idiopathic fevers. In phrenitis, as in pneumonia or enteritis, the fever is regulated by the state of the local affection—and the pulse harmonizes with that in the other phlegmasiæ. In *phrenitis*, there are very generally the local signs of cerebral inflammation, in addition to the general phenomena of fever.—In *idiopathic fever* the symptoms of local inflammation are seldom, comparatively speaking, present; for mere head-ache, or even delirium, is no proof of inflammation of the brain. There are scarcely any of the phlegmasiæ in which head-ache and evening delirium are not present, when the symptomatic fever runs high. In *idiopathic fever*, after the period of excitement, of greater or less duration, a stage of debility or collapse ensues, which is often of much longer duration than the previous one of excitement. This is not the case in true phrenitis. When the inflammation is subdued, nothing remains but a degree of weakness corresponding with the violence of the inflammation and the effects of the remedies. The termination of the two diseases, when fatal, is very different. In fever, Dr. Clutterbuck himself acknowledges, the delirium often subsides before death, and the sensorial functions are regularly performed. Do we ever see such a restoration of function in fatal inflammation of other viscera, as of the lungs, intestines, &c.? No, verily. But the inevitable rock on which every theory must be wrecked, which assigns local inflammation as the cause of the phenomena, is the *periodicity* of fever. Intermittent fever is the original and the purest type of all, in which the three stages are exquisitely delineated—and yet this fever will leave, every second day, a clear apyrectic period, for months in succession. No sophistry can torture this into inflammation of any organ. This last is a disease which cannot come and go in this manner, without leaving symptoms of its previous existence in the living, and demonstrable evidence in the parts after death. *Irritation* indeed, or any morbid condition which only reveals itself by pain or other phenomena, while itself is invisible, may be periodical; but inflammation is not one of those imperceptible or untangible agents. It is visible—it is an actual change of structure, and we cannot admit its existence, without ocular proofs. These proofs then, we think, Dr. Clutterbuck has failed to produce, as far as living phenomena are concerned; and if we look to dissection, the prospect is still more gloomy for our author's theory. Dr. Clutterbuck, indeed, is so conscious of its instability, in point of pathology, that he labours hard to throw an air of discredit, not to say ridicule, on post mortem researches in fever. First, the delicate structure of the brain renders it "unfavourable for accurate examination"—then "it more quickly undergoes a change in its texture than almost any other organ."—But the best argument against dissection is this:—

"It is to be considered, that by far the greater number of demonstrations of the human body, given by anatomists in the schools, and from which our knowledge of the structure and appearance of the brain is principally derived, are of subjects destroyed by diseases, many of them, no doubt, of this very organ. And when we reflect, further, on the sources from which the anatomical theatres are chiefly supplied, namely, the most indigent classes of society, a considerable number of whom are daily cut off by fevers, it must appear highly probable, that what is considered and exhibited as the natural and healthy state of parts, is often in reality a diseased one, perhaps the immediate consequence of fever itself, and which we have not yet learned clearly to distinguish from the state of health." 198.

Really this is too much! Then we are to set aside, as nothing, the innumerable dissections which have been made of patients who, in their lives, presented the symptoms of encephalitis and meningitis, and who, after death, exhibited the most unequivocal effects of the inflammation, as regular and as well defined as the effects of inflammation in any other organ of the body! Are we really so ignorant as not to be able to distinguish brains exhibiting these appearances, from brains exhibiting no appearances of the kind? Can we not tell a dura mater that is pale from one that is red*—one that is thin from one that is thickened—can we not distinguish a dewy vapour between the membranes from a flood of serum or flakes of coagulable lymph—are we so blind as not to see the difference between a tunica arachnoidea as transparent as crystal and thinner than cobweb, and one that is as thick and as white as foolscap paper? Is hardness, softness, vascularity—suppuration itself, in the substance of the brain, nothing that can lead to any determinate idea, even when connected with a train of well-marked and carefully noted symptoms before death? Nothing! "We have no certain standard of health," says Dr. C. "to which we can refer them, as objects of comparison." Con-

* If the following be a fact well authenticated, the public demonstrator of anatomy alluded to ought to be publicly exposed, and turned out of his situation.

"The brain of a person dead of small-pox was exhibited before a number of students, in the ordinary course of demonstration, in the dissecting-room of a public teacher of anatomy. And although the blood-vessels in general were turgid, and the membranes in many parts suffused with blood, like the coats of an inflamed eye (appearances that are very commonly observed after small-pox, and, as far as my observation has yet gone, in greater or less degree, in fevers in general,) no notice was taken of circumstances so strongly indicating inflammation; and, of course, the students went away impressed with the idea, that what they had seen was the natural state of parts." —193.

This, we repeat, was unpardonable, and almost incredible. We hope the circumstance has been exaggerated by the person who communicated it to Dr. C.; for had he been present, he must have taken notice of such a piece of glaring pathological ignorance.

sidering the numerous and minute investigations, as to the state of the brain in fever, which have been made in all parts of Europe as well as in these Isles for many years past, we can hardly pardon Dr. Clutterbuck for *repeating* the following sarcastic remark after a lapse of twenty years. "I am inclined to doubt, indeed, from the inquiries I have made, whether half a dozen such dissections have *purposely* been instituted in this metropolis, within as many years last past." Is this not a libel on the Fever Hospital? Surely in that institution alone five times the number of brains have been *purposely* examined among the patients who die of fever there. Be this as it may, there is no lack of authentic documents respecting the appearances on dissection, in fever, as the readers of this Journal well know;—but—*the grapes are sour*! These dissections do any thing but prove the ingenious Doctor's theory, and, therefore, they are depreciated. But, notwithstanding this utter worthlessness of dissections, Dr. C. has taken good care to collect, from every point of the anatomical compass, all the more striking testimonies—*that are in his own favour*—leaving the drudgery of quoting the adverse testimonies, or the adverse portions of the same testimonies, to his opponents. This may be good in *law*—but it is very bad in *physic*. Thus, Dr. C. quotes the venerable Jackson, as far as relates to the appearances in the *brains* of those who died of yellow fever—but not one word as to the *other organs* of the body—and so on, from Bonetus down to our own times. But it is very obvious that our able author is far from satisfied with all this gorgeous display of cerebral *adversaria*, so studiously gleaned from the dead and the living.

"A host of other instances," says he, "might be adduced to prove, that fevers of all descriptions very frequently leave behind them visible topical affections of the brain, demonstrating the existence of previous inflammation in that organ. It is not, however, to be imagined, that the appearances now mentioned are to be found in every case of fever. The *essential* part of this, as of most other *primary* diseases, consists, not in the altered structure of parts, but in morbid *action*: change of *structure* is a remote effect, a consequence, merely, of the morbid action, and is what may or may not take place." 216.

Now, we maintain that inflammation *is change of structure*—and we perfectly agree with Dr. C. that this is—"a remote effect, a *consequence* merely of morbid action." Surely Dr. Clutterbuck will not attempt to prove that *morbid action* is inflammation—for if it is, then we have inflammation in every organ in the body, as he has shewn that all the functions are in a deranged—that is, a morbid state. This very passage destroys his theory. He is driven up into a corner, *even by him-*

*self*, where he is obliged to admit that there is often no other proof of inflammation in the brain than *derangement* of its function—a derangement common to *every* other organ in the body, during fever; but which he will not admit to be any proof of inflammation, except in the brain!

"In an organ of such importance in the animal economy, and which so materially influences the actions of other parts of the system, it is easily conceivable that such a degree of *derangement* may take place as even to prove fatal, without leaving behind it any *visible* traces: and in reality, such has *often* been the case." 208.

Thus Dr. Clutterbuck, not being able to substantiate his doctrine in the *visible* world, very ingeniously repairs to the *invisible*, forgetful of the scholastic axiom, "de non apparentibus et non existentibus eadem est ratio."—Did it never occur to Dr. C. if he was in search of truth, and not of materials for a theory, to ask himself this question—if the organization of the brain be so very delicate that its functions may be annihilated before there is time for any visible change in its structure, how is it that, in by far the majority of cases where we have reason to suspect inflammation, its ravages are clear as the sun at noon-day? If, indeed, life was cut off in the first onset of fever, there might be some reason for arguing that the sensorium was overpowered, and that there was not time for the production of organic changes; but when we see men linger out for 12, 14, or 21 days, with all the phenomena of idiopathic fever, and yet the brain unaltered in appearance, the conclusion is irresistible, in the unprejudiced mind, that the seat of inflammation was not in the brain. Do we see the phenomena of inflammation continue so long in the lungs, liver, or intestines, and on dissection these organs presenting no trace of alteration? No, truly. And surely organs of so much stronger a construction than the brain, ought to be able to withstand *derangement* of function, morbid action, or whatever other term Dr. C. may apply, for a longer period than the brain, without being altered in structure.

If Dr. C. in collecting evidence respecting the pathology of the brain in fever, has passed over the statements of adverse authors, and the adverse statements of the authors which he did quote, he comes upon them by a side-wind, in the shape of a rapid review of the opinions of ancients and moderns, in regard to the seat and nature of fever. It is very easy to conceive that their theories are demolished much in the same way that a dog would demolish a whole posse of mice—they are slaughtered, without remorse, and their then lacerated members thrown to the winds. The lives of a very few are spared with only the slight sacrifice of a limb or two. The first act of mercy which we notice is in the case of Mr. now Dr. Peiw, who, more than "20 years ago,"* broached a doctrine of fever bearing some analogy to Dr. Clutterbuck's. It was *congestion* of blood, however, in the brain—whereas, the doctrine is now inflammation. The late Dr. Home, of Edinburgh, is also spared, in consideration of a confession which he made on the bed of sickness, that he had had an inflammation of the cortical part of the brain in the shape of a low nervous fever. But a circumstance has here surprised us extremely. Dr. Clutterbuck has never once alluded to Ploucquet, whose doctrine of fever, as published at Tubingen, in two theses, 1800 and 1801, is so exactly that of our author, that Dr. Beddoes has filled sixteen pages with a parallel of the particular propositions in which the doctrine is propounded, and where the coincidence is such that, as Dr. Beddoes observes, "the English may often seem a diffuse translation of the Tubingen Professor."—Was this proper? was it candid to quote a few authors, whose doctrines nearly approached, and went to the *support* of his own; but to draw the veil of oblivion over Ploucquet's, which completely *anticipated* it? This is a question which Dr. C. may find it difficult to answer in a manner satisfactory to others.

Before quitting the subject of pathology, we may advert to the way in which our author endeavours to disentangle himself of the awkward fact, that the traces of inflammation are as often—nay, oftener found in the viscera of the thorax and abdomen than in the brain. Nothing is more easy than the explanation.

"One obvious cause of obscurity with regard to the *primary* seat of disease in fever, and a reason why dissection has failed to point it out, is that patients are often cut off by the *consequences* of fever, rather than by the fever itself; by inflammation of other organs coming on during its course, modifying the character of the original disease, and ending in disorganization of the part *secondarily* affected." 217.

And why, we ask, may not the inflammation of the brain come on *secondarily*, as well as that of other organs? The only reason which Dr. C. can possibly give is, that the first phenomena of fever are shewn in the nervous system. But we say again that the *first phenomena* of fever are *not* the pheno-

* This pamphlet was published in 1785, which is just 41 years ago, and yet Dr. C. allows the expression to stand as it was first written in 1805. Surely this is misleading the public, and not doing the duty of revision in new Editions of works.

mena of *inflammation*, but of depression. The symptoms of inflammation, when they do occur, always rise during the *re-action* which succeeds, and *then*, they do not always shew themselves *first* in the brain, but often in other organs, and often not at all in the head. We cannot, therefore, agree with Dr. Clutterbuck—" that the symptoms of fever are the symptoms of inflamed brain"—and " that fever and inflammation of the brain are identical affections." p. 220. If we did, we must admit that there is inflammation of the brain in every case of small-pox, measles,—nay of compound fractures or gun-shot wounds—for in *all these* we have *all* the phenomena of idiopathic fever, when the latter is completely formed.

Dr. Clutterbuck rests a good deal, for support of his doctrine, on the nature and operation of the *remote causes* of fever. Whether we confine these, as Cullen did, to marsh and human effluvia, or extend them, as most modern observers do, to a considerable number of agents, as atmospheric changes, intemperance, agitations of mind, &c. we see no reason to suppose that all or any of these causes should tend exclusively to produce inflammation of the brain. We grant, indeed, that most of the febrific agents tend to disturb the function of the brain and nervous system—but what morbid agent does not the same thing? Why should the effluvium from a marsh inflame the brain? It is not first applied to that organ, and the symptoms of the fever which follows as well as the appearances on dissection, shew other organs, as the stomach, bowels, liver, and lungs, more frequently affected than the head. For proofs of this, we may refer to all the best modern writers on marsh fevers. Dr. C. seems conscious that he has tender ground to tread on in etiology. He acknowledges that—" of some of the causes of fever, it is difficult to assign the mode of acting;" but of others, he thinks, the mode is manifest enough—and very appropriate for his doctrine. He, therefore, singles out *intoxication*, " which when carried to a great excess, is often succeeded on the following day by head-ache, increased heat, and other symptoms scarcely, if at all distinguishable from fever generated from other sources." It is rather unfortunate for our author's theory, that this fit of intoxication does not once in 999 times produce a fever—or any other effects that last more than twenty-four or forty-eight hours after the debauch. It is still more unfortunate for the illustration, that a pint of wine (as Bacchanalians well know) will generally put an end to this fever—or this inflammation of the brain, on the evening of the day succeeding the debauch. The fact is, that the head-ache and other febrile symptoms, *post inebrietatem*, are more dependent on the stomach than on any other organ—and when the atony of this organ is moderately stimulated the next day, the whole of the nervous symptoms vanish.

In respect to mental causes, every body knows the influence of the mind over the functions of the body; but whether we look at the case quoted by our author from Van Swieten, "of a girl who, when in health, being terrified at the unexpected sight of a dormouse, fell immediately into a quartan ague, which continued to recur for a whole winter;"—or the other more classic case recorded by Pliny, of "Quintus Fabius Maximus, who was cured of a *quartan ague* instantly on entering into battle with the Allobroges and Averni," we can see nothing in either of them to support the doctrine, that fever is inflammation of the brain. If the sight of the dormouse did produce the quartan fever—and if fever be inflammation of the brain, how the devil did it happen that this inflammation rested from its labours—or, at all events, shewed no proofs of its existence during two days out of three for a whole winter? On the other hand, how did the conflict and excitement of a battle cure inflammation of the brain in Quintus Fabius Maximus? In our humble apprehensions, both these cases, allowing them to be authentic, make directly against the doctrine in question, by shewing the facility with which the phenomena of fever may be raised or removed by the *same* cause. This would be very unlikely to happen if the case was phrenitis!

We shall notice only one more etiological illustration brought forward in support of Dr. C's doctrine. He says, it is remarked that maniacs are less liable to fever than other people, probably from "a defective or morbid condition of the brain," which renders them insensible to a variety of impressions, both internal and external. Of this immunity from fever we have some doubts—but if a morbid condition of brain be a protection against inflammation of that organ, it is contrary to all analogy at least—for nothing is more certain, than that the morbid condition of any other organ in the body proves a powerful predisposition to inflammatory action, whenever the individual is exposed to the proper causes.

We have now only to speak of the treatment of fever in relation to the theory of our author. Here too, we clearly perceive the difficulties which Dr. Clutterbuck had to contend with, in order to draw any thing like support to his doctrine from the important subject of therapeutics. Dr. C. foresaw that this would probably be made the touchstone of his theory, and he has done all that human ingenuity could devise to strengthen this the weakest side of his camp. He sets out with rather a disheartening sentiment, namely, that "it is hardly to be expected that any theory, however just in its principles, will, at once, materially improve the cure of fever, or detract much from its danger and fatality." But he observes, and justly, that a true theory must be advantageous, inasmuch as it will keep us from doing harm where we cannot do good. Speaking of the routine practice in fever, Dr. C. makes the following sarcastic remark, in which we perfectly agree with him.

"For want of a distinct and intelligible object in view, on the part of the practitioner, and a desire of being active, the patient is made to undergo the whole routine of medical treatment; being, in turn, *bled, blistered, vomited, purged, and sweated;* and afterwards *stimulated* in various ways; one means being resorted to after another, for little other reason, as it would seem, than because the former had failed. A recovery, no doubt, often takes place, even under these circumstances, but it is probably less to be ascribed to art than to the powers of resistance of the constitution, the *vis conservatrix naturæ;* which is often not only an overmatch for the disease, but for the treatment pursued also." 275.

As it is a well known fact that but few fevers, comparatively speaking, terminate fatally without leaving traces of local inflammation in some organ or other; so we are willing to allow our author all the support which the above knowledge may give to his doctrine, on account of the antiphlogistic mode of treatment so generally adopted in fevers during the present century. This antiphlogistic treatment is acknowledged to have been carried a great deal too far—and there is now some degree of recoil or reaction in the public opinion; but, granting that the practice is infinitely preferable to the Brunonian practice which preceded it, still we must remember that, were Dr. Clutterbuck's theory correct, it is next to impossible that one in ten of the fever patients treated with bark, wine, and opium, could have survived—whereas we know, from the most authentic records, that they did recover—and not in a much less proportion than in the present day—probably for the reason advanced by himself in the last quotation which we have introduced. It is in vain for Dr. Clutterbuck to tell us that there are certain inflammations, as acute rheumatism and erysipelas, which are not to be cured by active bleeding—and that the brain may have its peculiarities, in this respect, and may not bear depletion when inflamed. If fever be a *specific inflammation* of the brain, differing in its nature and treatment from common inflammation, then Dr. Clutterbuck's labours have been nearly in vain, for we are left just as much in the dark as ever.

It is quite needless, we imagine, to follow Dr. C. in his quotations from authors who have recommended blood-letting in fevers. They have, almost all, done so to *prevent* the occurrence of inflammation, or to check it when it had commenced in some organ, *during the fever*. In this they were perfectly right—and it is on this principle that all observant practitioners of the present day employ the lancet in fever. But the very circumstance of fevers being cured, in great numbers, by the most opposite plans, and especially by bark, wine, and opium, must carry conviction to every mind not warped by a favourite theory, that fever is not in its essence a local inflammation—and that still less is it a local inflammation of the brain.

We deem it unnecessary to dwell longer on the subject of treatment, since every practitioner, of any experience, will immediately see that Dr. Clutterbuck's theory will not apply to practice at the bed-side of sickness—at least as far as blood-letting is concerned. The following passage, alone, we think, will shew how inapplicable is the theory to therapeutics. "As a general principle, it may be observed, that the more the *sensorial* functions are disordered and oppressed, the less likely will blood-letting be to prove effectual."—p. 351. If this be the case, how is the practitioner to be guided? Let us apply this reasoning to any other organ inflamed—say the lungs. "The more the *pulmonary* functions are disordered and oppressed, the less likely will blood-letting be to prove effectual!!" Here we see how bewildering is the doctrine, when brought to bear on actual practice! In fine, we are convinced from a long and attentive observation of the phenomena of fevers, as well as study of their pathology, that the only safe theory to bring into the bed-room is the belief or opinion that fever is a general disorder of the system, which may or may not produce local inflammation, according to the condition of those organs, or the idiosyncrasy of the patient—and consequently, that depletion is to be proportioned to the degree of general excitement—always remembering that there is a stage of debility to succeed—and that we are not to deplete as if the general excitement were the consequence of local inflammation. The next thing to do is, to watch *where* the local inflammation falls, if it takes place at all—and meet it by local rather than by general means. This comprises the whole plan of indications in the stage of reaction. When the powers flag and the constitution begins to run down, then it is that the young champion for local inflammation feels the treachery of the doctrine on which he reclined, and will often have to deplore the freedom with which he acted previously. The great difficulty is to hit the happy point of safety, in neither depleting too much nor too little, in the early stage of fever, since error on either side leads nearly to the same bad consequences in the end. A violent and uncontrolled excitement cannot go on long without producing such disorder in some vital organ as will render the winding up of the fever doubtful or dangerous. A too great reduction of the powers of nature, at ever so early a period of the disease, will lead to a similar result.

We give Dr. Clutterbuck great credit for supporting his doctrine with all the force which ingenuity, erudition, study, and long experience could bring to bear on the point. The doctrine, indeed, is advocated with such skill and plausibility, that it is almost certain to captivate the judgment of the young and inexperienced. Thirty years ago we would have embraced it without hesitation, but we think that ere now time and observation would have cured us of the doctrine. We think it is a doctrine that is dangerous—though perhaps not so much so as Brunonianism. But it is very seductive to the young mind—and on this account we have taken more pains to point out its fallacies than we otherwise should have done. With proper precautions, its perusal will afford the practitioner as well as the student, a fund of information on all points respecting the etiology, symptomatology, pathology, and treatment of fever.

---

But it is now time to turn to M. Broussais, whose writings we have often noticed, and whose doctrines we have frequently criticised, as our readers well know. In the second number of our Quarterly Series, (September, 1820) we gave an account of Broussais' Observations on Peritoneal Inflammation. In the succeeding Number of the same Series, (September, 1820) we exhibited the Professor's Treatise on the Mucous Membrane of the Lungs—in No. 5, (June 1821) we reviewed M. Broussais on Affections of the Mucous Membrane of the Digestive Organs—and in a late Number of the present Series (No. 6, for October, 1825) we took occasion, while reviewing Montfalcon on Marshes, to introduce some strictures on M. Broussais' New Physiological Doctrine of Fever. Still we have never yet given any thing like a full view of this now celebrated doctrine, which has made a prodigious number of converts on the Continent, but not without a most spirited—indeed violent opposition. M. Broussais has been infinitely more active than Dr. Clutterbuck in the support and propagation of his doctrine. He has not suffered twenty years to elapse between his first and his second publication, as our phlegmatic countryman has done. No, No. He and his numerous disciples never let a year, or hardly a month pass, without urging their cause, *vi et armis*. They seem to have adopted the plan of Mahomet—" propagate the doctrine of Islamism by persuasion—but if persuasion fail, use the sword." They seem also to have taken a leaf from other churches than that of the Prophet. " The *end* shall justify the *means*." Fanaticism and enthusiasm are not confined to religion and politics. They mix themselves with all sciences that have not mathematical exactness for their basis—and it is hardly necessary to say that medicine is not *yet* a science of the latter class, though this is positively insisted on by the disciples of Broussais. " Medicine is become a science founded on *invariable* principles."—Preface, p. ix. Would to God that this was the case ! Periodical criticism would then indeed cease to exist, but the public would be infinite gainers by a uniformity in the practice of medicine. This epoch, however, is not yet arrived ; but it may not be lost time to give the English reader some idea of this new system which is said already to have attained the height of a demonstrable science. It would perhaps be more regular to give an analysis of M. Broussais' professed work, the " Examen des Doctrines Medicales," rather than that of his disciple, the anonymous author of the " Conversations ;" but, on examination, we find that the doctrines of Broussais are fairly embodied in the *latter*, with some new touches—doubtless from Broussais himself, who is the grand mover in the medical drama now acting on the Continent. We shall have occasion, however, to advert to the " Examen" itself, in the course of this article—particularly that portion of it which is dedicated to the medical literature of this country, and where we shall have a crow to pluck with brother Broussais.

The disciple of the new doctrine, introduces the subject by a " personal narrative," of which we shall take very little notice, except where it bears immediately on the subject. Having composed and sustained his thesis in Paris, and obtained the Doctorate, he retired to his native town, where his first patients were, as usual, the members of his own family. His father became ill, and the following extracts will serve well to open the business on which we are entering.

" For several weeks, my father complained of a slight derangement in his health. The emotion caused by my return, excited occurrences that interrupted our joy. His head became painful ; he complained of a sensation of extraordinary fatigue in his limbs, and particularly in the regions of the back and loins ; fever manifested itself, though moderate ; his tongue appeared covered with a thick foulness, yellowish in its centre, whilst it was red in its circumference and at the extremity. He felt acute pains at the pit of the stomach ; he had a bitter taste in the mouth, and a continual inclination to vomit; nor could he divest himself of a fatal presentiment, affirming, that my return would inflict upon him the stroke of death; but this kind father consoled himself in the reflexion, that he would leave me behind with an honourable existence, and worthy of the sacrifices he had made for me. This conversation was heart-rending; but the confidence I had in my extensive information straightway encouraged me. It is but a deranged stomach, *embarras gastrique*,* with fever, said I; to-morrow you shall take an emetic, and all will be well. The night was restless, and the emetic, which I administered myself largely, produced abundant evacuations. A visible amendment was perceived after its effects; the tongue became more clear, and red in its whole surface. I confidently triumphed; but towards evening the symptoms redoubled their intensity; the fever acquired such a degree of violence, that my hand could scarcely support the heat of the skin, especially at the epigastric region; the beating of the pulse was accelerated; the tongue, from the scarlet red became brownish, contracted, pointed, and dry; a parching thirst succeeded to the bilious and clammy taste. The sensibility at the pit of the stomach appeared more acute, and that of the limbs was so excessive as to force cries from my father, who no longer dared to use any motion.

"This exasperation did not much alarm me: it is a gastric fever that declares itself, said I to my father; and you are very fortunate in having been relieved of your *saburra*, for it might have assumed an adynamic character. What alarms you at present is no more than the inevitable augmentation in these disorders; but to-morrow you will be better, and the fever will terminate favourably the seventh day: in the mean time drink veal broth to refresh yourself, tamarinds with whey, in order to keep the body open, and arm yourself with courage and hope.

"The third day, the fever, in place of subsiding, as I had predicted, was aggravated; the pain in the head was excruciating; the most intolerable thirst was rather exasperated than appeased by liquids, which now went strongly against the stomach of the patient. The face was flushed, and delirium at intervals perceptible.

"These symptoms alarmed me. The head is threatened with a congestion, said I to myself; the fever is not only gastric, but puts on the character of the ardent fever, or *causus* of Hippocrates. Let us immediately try bleeding in the foot; but let this be moderate, for I observe some tendency to ataxia; and a too copious loss of blood may cause this to degenerate into adynamic fever.

"The patient was bled, and was relieved, but he was near fainting; and this slight accident having confirmed my fears, I thought it my duty to raise the powers a little, by administering a few cups of a weak vinous lemonade. Scarcely had a few doses of this fresh specific been

---

* "*Embarras gastrique,* the first species of the second order of febrile diseases of the French nosologists; the symptoms are, head-ache, anorexia, bitter taste in the mouth, a yellowish or whitish coat on the tongue, nausea, particularly in the morning, pain at the epigastrium.—*Note of the Translator.*"

given, when the fever revived, not with a pulse large and full as before, but with pulsations shrunk, and as if convulsive. The tongue put on a brownish colour. My father no longer complained of any pain, but he became totally delirious. His strength was fallen, his limbs agitated by those movements which we call *subsultus tendinum.* I saw in this stage the combined symptoms of ataxa-adynamic fever, or, to speak the old *jargon*, putrid malignant, and I was preparing to inflict the last stroke on my unfortunate father, by administering the pure wine of cinchona, camphor, musk, and the serpentaria of Virginia, when an occurrence, as fortunate as unforeseen, preserved me from the parricide I was about to commit." 7.

Our author was prevented from committing parricide by the lucky interposition of an old fellow student who, while in Paris, had become a convert to the new doctrine. His friend declared that the disorder was *made* by the mode of treatment, and that it might have been checked in the beginning by leeching the epigastrium. The son yielded, and the friend stood physician.

"Fifty leeches were applied on this same evening; and their punctures bled abundantly during the whole night. In proportion as the blood flowed, my father recovered his intellect and strength; he repeated over and over, I am saved! I was overpowered with joy. On the morning of the fifth day, the fever was entirely gone, but the weakness alarmed me. I wished the patient to take some beef-broth; but my friend objected to it, assuring me that this light restorative would be sufficient to renew the whole mischief. I yielded. We left the patient to enjoy a profound sleep during the greater part of the day. But judge of my surprise, when, at his awaking, he asked for food, alledging that he was perfectly well, able to get up, and had no other illness than weakness and hunger. Still nothing was given him but a little lemonade, after which he again fell asleep. The next day he had some broth; the day after beef broth, and he got up. Calculating from this time, he only complained of extreme hunger, which was satisfied with caution; and his convalescence was so rapid, that four days afterwards there was no longer an appearance of his having been indisposed." 9.

The conclusions which the novitiate was obliged to draw from this example were—that the derangement of stomach, with fever, was the first degree of inflammation of that organ—that this was exasperated by the emetic—that veal broth and whey were too stimulating for an inflamed membrane—that the bleeding from the foot was not copious enough, and was counteracted by the vinous lemonade—that one local bleeding near the seat of the complaint "is an hundred times more efficacious than bleeding in the large vessels"—that the inflammation must be left to subside entirely after the bleedings, before food is given. Finally, he candidly concluded that he knew nothing of medicine—and that he must hasten back to study under the grand master.

The Savant here makes a remark, which is far from being satisfactorily answered—namely, that, as emetics often cut off fevers at the beginning, it is probable that they do not all commence with gastritis. The answer is—"because the evacuation of bile, mucus, and perspiration carries off the irritation of this organ," in some cases; but "he who prescribes an emetic, runs the risk of doubling the inflammation of the stomach, in case he does not remove it." In hot climates, and especially in the yellow fever of the West Indies, we think the above reasoning is not inapplicable; but it is far from applying generally to the fevers of Europe.

*Savant.* Then all physicians, hitherto, have been in error respecting the nature of fever. *Phys.* All. They wanted the principal facts on which science is erected. *Savant.* You are presumptuous—but not the only arrogant man in medicine. "The annals of this science attest the existence of a multitude of pretenders who exclaimed, in their ridiculous enthusiasm,—'I have the true science—burn all books—listen to me.' Paracelsus, Vanhelmont, Brown, and a great many others have held this language."

*Physician.* Is it impossible to discover that which has not yet been discovered? If I prove to you that we have discovered important truths, will you reject them because they happen to be of our time? I will proceed to the development of my proofs. You have just seen a fever, in the first stage gastric or bilious, afterwards putrid or malignant, arrested in its first period, by the knowledge of a fact unknown to the ancients. This fact is that the fever depends on inflammation of the digestive organs—a circumstance of which the ancients must have been ignorant, for they did not arrest these fevers, but let them proceed, counting their days, and waiting for a crisis—or else they exasperated them by giving injurious medicines. Suppose, in a village, town, or hospital, a hundred disorders beginning like my father's. If these are all met the first day by leeching the epigastrium, they will be stopped. If they are treated, as I treated my father, they will go on, "and a *moiety* at least of them will die." Others will be prolonged, and leave to their victims a precarious health. Some will be cured, in spite of improper treatment, by the efforts of Nature, &c. In the first mode of treatment there will be no epidemic—in the second, there will be an alarming one. The sick, if together, will become a focus of infection, endangering the health of those who are obliged to approach them.

*Savant.* Prove to me by the *symptoms* that what are called idiopathic fevers are nothing else than gastro-enteritis.

*Physician.* With all my heart.

"The bilious or gastric fever is only a gastro-enteritis, in a person whose digestive canal, much irritated, renders the locomotive muscles painful, and the bilious secretion very abundant. You have seen the symptoms and treatment of the disorder in my father. The mucous fever is the same disorder in a lymphatic subject, and in one whose digestive canal secretes a quantity of that mucosity called *slime;* it is characterized, according to authors, by a foul and clammy mouth, by aphtha, salivation, mucous vomitings, or stools of the same nature, pustules and scabs equally mucous, and by the slowness of its progress, which is only because the phlegmasia was improperly treated in its commencement. But it is now known that mucous secretions by the mouth or inferior channels, accompanied by fever, inappetence, thirst, pain or constraint in the digestive canal, pain in the head, or a sensation of fatigue and weakness in the limbs, indicate inflammation of the membrane called mucous, which lines the interior of the digestive canal from the mouth to the podex. The expression, ardent fever, imports a very high degree of heat and fever in these same affections. The adynamic fever, in which, we are told, the preceding terminate, is in reality nothing more than the gastro-enteritis, arrived at such a degree of intensity, that the strength diminishes, the intellectual faculties are blunted (which causes a sort of heaviness, called stupor,) the tongue becomes brown, the mouth is lined with a blackish coat or crust; but this dark colour of the mouth was preceded, in the commencement, by a bright red; the black mucous has been white, yellow, or grey, on the first days; and all this change has taken place because the inflammation was not stopped in the beginning. This fact is so certain, that one seasonable application of leeches removes the stupor in a few hours, forces back the brown colour of the tongue towards a shining red, cleanses the mouth of this mucous blackness, which rendered it, as we say, fuliginous, and re-establishes the force in the muscular system. The term putrid fever only indicates the fetidity of the breath, perspirations, and stools, that are joined to the preceding phenomena or symptoms. The malignant, or cerebral fever, is but the irritation of the brain, added by sympathy to the gastric inflammation, which produces the pretended bilious, mucous, and putrid fevers. For when the brain is primarily inflamed, this state is designated by the terms frenzy, *arachnitis*, or *encephalitis;* but it may so happen, that the irritation of the brain, although secondary, may rise to the degree of a true inflammation; or that of the digestive canal may be developed consecutively to the *encephalitis*, where the digestive canal is not very painful, or where the bile and mucosity do not abound; very intense, it approaches to the ardent fever: but it is often the first stage of all the others. So we are told by authors, that, if it does not terminate in a few days, it runs into gastric, putrid, or malignant fever; which infers, that the inflammation of the digestive canal, at first mild, rises to a degree that causes debility, fetidity, or else is complicated with irritation of the brain.

"These are the essential fevers of authors; other inflammations may be associated with them, but this is not what has been pretended to be marked by the expression, essential fever; for they had terms to indicate phlegmasia of the lungs, heart, liver, and other textures. The term, essential fever, with authors, only designed gastro-enteritis; which not being known, made them believe in the existence of a general affection. They were ignorant that the fever was maintained by the phlegmasia. They could not reconcile it to that which they knew; they considered it then as independent of any organ in particular; as existing of itself; in a word, as essential." 21.

After animadverting on the conflicting opinions respecting the treatment of fever, the disciple of Broussais expounds the "clear and precise" doctrine which guides the new school. Employ, says he, neither emetics nor cathartics at the commencement—if there be extreme plethora, bleed generally, though in most cases it may be dispensed with—the number of leeches must be proportioned to the strength of the patient, and the violence of the disease—the blood from the orifices should be permitted to flow, and especial care should be taken to enforce abstinence from nutritive drinks after the operation of the leeches. If the inflammation does not yield to the first application, it is to be repeated, as long as the patient is not exhausted—but if he was affected, previously to the attack, with chronic inflammation, and is now much fallen away, the curative means ought to be limited to emollients. It is with lemonade, currant jelly-water, or even pure water, to the exclusion of all soups or broths, that the disease is to be treated. At the same time the practitioner may employ lenitive enemata, the pediluvium, cold water or ice to the epigastrium or the head in hot weather and where there is no tendency to inflammation of the lungs. With these means calmly wait, till nature conducts to a cure.

From various causes the disease is sometimes protracted beyond a month; but this is nothing to what takes place on the Brunonian plan. "The irritation which they cause in the digestive organs, as soon as the patient's strength begins to fail, hurries on the work of death, in the midst of convulsions and delirium. Sometimes, however, there are those who resist, and if they are not extricated by a violent crisis, they remain in a state of languor much more than a hundred days; for their health continues precarious for a long time."

In prolonged cases, treated on the plan of Broussais, the patients suffer so little, that many physicians seeing them quite free from pain in the abdomen, have come to the erroneous conclusion that the fever may continue independent of its original local cause, being " ignorant of the fact, that the inflammation of the mucous membrane of the digestive canal is rarely painful —that in order to be known, it does not require the *local* sensibility :—the sense of obtuse pain in the limbs, the inaptitude for exercise, the frequency of the pulse, the burning heat of the skin, pain in the head, are sufficient to characterize it, when combined with redness of the tongue, inappetence, thirst, a higher degree of heat on the belly than on other parts, and, for a much stronger reason, the fuliginous appearance of the mouth, brown colour of the tongue, and stupor."

" They did not understand the mode of sensibility of the digestive canal, and knew not that this irritation is rather recognized by the influence it exercises over the other organs, and by the painful sensation developed in these, than by its own pains. But an attentive disciple of the physiological doctrine is not ignorant of these peculiarities. He recognizes the gastro-enteritis, without requiring to press forcibly the abdomen of a patient to cause pain ; and on the slightest indication he at once attacks the disease, removes it, and anticipates the explosion of all those pretended fevers, which have been the torment of physicians in past ages." 27.

*Savant.* I can comprehend how a fever may be arrested, in the beginning, by bleeding ; but when it is arrived at the state of adynamia or weakness, the antiphlogistic treatment would seem improper.

*Physiologist.* This is an error. *Debility* is apparent in the muscular system, because *power* is concentrated in the viscera, as proved by the excessive heat, reflected thence on the skin—extreme quickness of pulse—and, by the promptitude with which the muscular powers are re-established when blood is withdrawn. There are few physicians who do not begin the treatment of fever by antiphlogistics ; but, as soon as they perceive debility, they have recourse to stimulants, the consequence of which is, the revival of the phlegmasia, ulceration of the mucous membrane, and a long protracted cure, if life be saved at all.

*Savant.* Can ulceration of the digestive canal in fever be cured then ?

*Phys.* Yes. Nature effects a cure in a few weeks, if the disease is not exasperated by stimulants, provided the patient is not too much exhausted; for, when the patient at length sinks, there are found in the intestines numerous traces of cicatrized ulcers, which proves that, had the patient possessed force to resist for a longer period, life would have been preserved.

*Savant*. Therefore his strength should be supported to prevent his sinking.

*Phys*. I grant it. But it is with mucilaginous, gummy, and saccarine beverages, with a small proportion of milk, that this ought to be done. The weakest chicken-broth is sometimes sufficient to exasperate the inflammation, and its consequence, the fever. What, then, must be the effects of strong soups, wine, bark, and incendiary drugs ?

*Savant*. But it is generally allowed that convalescence is very tardy after the treatment you advocate.

*Phys*. This we deny. It is only a malicious report of our enemies.

The above is the theory, and also the practice, of the new school. It is infinitely more simple and easy than even Brunonianism. Any student, of common talents, may learn it in a fortnight, and thus become a physician superior to all others, ancient or modern, those of the new school excepted. For proofs of the truth of this doctrine, and the success of the practice, M. Broussais refers you to the phenomena of fever at the bed-side—the *post-mortem* appearances on dissection—and the clinical experience of the VAL DE GRACE, or any other place where the treatment is put in force. But the course of the finest theory, like that of the truest love, "never did run smooth." Those INTERMITTENT fevers which have proved a stumbling-block to our countryman, of cerebral celebrity, are rather difficult of digestion for him of the gastric doctrine. But what will not ingenuity conquer ? We must recollect, say the disciples of Broussais, that the irritation which raises motion, sensation, and attracts the fluids to any part of the body, is not always such as to follow up its destructive work to the point of disorganization. When it has these last characters, it is called inflammation.

"But sometimes the irritation is moveable; so that after having acted a certain time on one membrane, it deserts it, to make its appearance in another, or else it returns constantly to the same point; after having left it in a state analogous to that of health or a primitive state. Well then, these changeable irritations going from one place to another, or affecting always the same place, differ but very little from fixed irritations; they are produced by the same cause, they yield to the same curative means, and, if they are exasperated by stimulants, they intermix or complicate entirely with them.

"P. These irritations, which I told you were susceptible of being transported from one texture to another, appear under different names in the authors that have treated on them. To give you an idea, I select the gout and rheumatism; you know that physicians consider them as inflammations, notwithstanding their great mobility, because, during the stay these make in an articulation, there is observed in it redness, heat, and pain. Well, then; this changeable irritation sometimes becomes fixed in one of the parts which it was in the habit of leaving, or in a new situation; and from that time acts as if it had never been moveable. Moveable or fixed, the gouty irritation depends always on the same causes; moveable or fixed, it ought to be combated by the same means. You thus see, that there exists a great analogy between fixed and moveable irritations." 174.

These considerations prepare us for the solution of the problem respecting intermittent fevers. Do we not see men who have annual attacks of gout, or erysipelas, and, in the intervals, they are well? Why then should we not admit a *tertian* as well as an *annual* inflammation? The plus or minus of interval makes no real difference. An intermittent fever then is a tertian or quartan irritation or inflammation of the mucous membrane, which is of the moveable or migratory kind. It leaves its place at a certain hour, every second or third day, and, instead of visiting any other part of the body, it prefers returning, at a certain hour, to its old habitation. What can be more simple, clear, and convincing, than this ingenious explanation? There is only one little difficulty remaining, and this will be easily got over. Cinchona has (though it *ought* not to do so) cured agues. That may be, says Broussais; but the exception is far better than the general rule. It is a positive fact that bark has changed an intermittent into a continued fever, which is proof sufficient that the cause was an irritation or inflammation in the stomach. As for the pretended cures by bark, it will be found that almost all the patients so cured are "cachectic, with large spleens, enormous livers, oppression, cough—or, otherwise, they are in a state of dropsy or scurvy." "Now, all these infirmities are the produce of chronic inflammations of the viscera, which the stimulant action of the cinchona has substituted for the periodical irritation at the commencement." Q. E. D. To such irresistible arguments as these it would be vain for us to attempt a reply. It is somewhat curious, however, that the two leaders on opposite sides of the channel, should never, even by accident, make the slightest mention of each other's names, or allusion to each other's doctrines. This is the more remarkable, as Dr. Clutterbuck published his first edition some thirteen years before Broussais—while, on the other hand, the works of Broussais were thirteen years before the public, when Dr. C. published his second edition. From all that can be gleaned from their writings, they do not appear to be aware of each others' existence, though not 400 miles apart! And yet

M. Broussais, in his "Examen," surveys, with a critical eye, the state of medical literature in Great Britain, up to the year 1821—not, indeed, entering into combat with the leading writers of these isles, from whom he might have collected the sense of the profession, on most points, both of theory and practice; but, making war principally—"on rats and mice, and such small deer," as he found collected together in the "BIBLIOTHÉQUE BRITANNIQUE" extracted from the lucubrations of a few minor and unknown contributors of *original cases* in the Medical Journals of this country!—The list of English authorities given by TOMMASINI, a few years ago, and so admirably exposed by our friend Clarke of the eternal city, was a paragon of pure medical history, compared with the catalogue of English worthies which has been distributed over the Continent, with comments, by brother Broussais—who, by the bye, cannot read one word of English! Dr. Bowes, Dr. Clyston, Dr. Bigsby, Dr. Newnham (they are all doctors here) Dr. Kinglake, Dr. Rogers, &c. have reason to complain of the ingratitude of their countrymen, in not awarding them a higher niche in the temple of fame at home;—but they have the consolation of knowing that they are the true representatives of the British profession wherever the works of Broussais have travelled on the Continent! True it is, they are held up to ridicule by this learned professor, who frisks round each of them as a cat would frisk round a mouse—and at length swallows them at a mouthful, when he is tired of laughing at them! But still, any thing is better than neglect.

Yet it may not be quite unprofitable to hear what a man of Broussais' reputation thinks and says of us, in his review of theories and practices of different ages and nations—none of whom, not even the French, he spares—so that we cannot complain of his partiality, whatever we may do of his severity.

The English, says he, affect to despise Brunonianism, and the greater number preserve strict silence respecting the works and discoveries of their Continental neighbours! Yet they secretly profit by all—though their practice is a strange medley—and consists principally of empiricism. They are inclined towards the humoral pathology; but almost all of them talk in a tone of inspiration, as if they had invented the science, without giving themselves any trouble to prove their assertions. Some of them say they can cure all diseases by purgation—but most of them join blood-letting and opium. These three means, with a few specifics, constitute almost the whole of their *methodus medendi*.

When they have acute diseases to treat, they seldom enquire into their seat or nature. They draw their lancets first, and then pour in cathartics. Calomel is a great favourite with them. They give it in almost all diseases—*even in yellow fever!!* Some of the English join gamboge and colocynth with their calomel as a purgative. They have no patience to wait the efforts of Nature. Every visit is distinguished by a new prescription, containing the most potent drugs. Delirium, colic, convulsions—nothing stops their hands! Blood-letting, purgatives, opium, oil of turpentine; tinctures, aromatics, strong wines, all testify to the sick and to the assistants the prodigious and inexhaustible resources of the doctor! Like Brown, whom they imitate, they leave nothing to Nature who they consider incapable of any salutary effort.—Art must do all, in their system—and in truth, it is most efficacious; for this furious practice does not effect a crisis, it soon puts an end to the patient by disorganizations of the principal viscera, which the doctor beholds with astonishment after death, never dreaming that he occasioned them all himself—a sure proof of the profound ignorance in which the English are plunged, touching the mechanism, and the functions of the viscera, or the application of physiology to the treatment of their diseases!* It is thus that we can explain the practice of Dr. Newnham, who tortured with emetics and purgatives a patient affected with gastric irritation, till he produced a cancer, which he only discovered on dissection. "Qui tourmente par des emetiques et des purgatifs une personne affecté d'irritation gastrique, et produit par cette perturbation, un cancer qu'il ne reconnait qu'a l'ouverture du cadavre."

And yet, we would recommend to some of our Abernethian disciples an attentive perusal of the following passage—"fas est et ab hoste doceri."

"It would be difficult to convey an idea of the extravagant passion for purgation which obtains in England, especially in chronic complaints. In this respect the faculty are in perfect accordance with the prejudices

* M. Broussais is not aware that we are obliged to draw all our finest specimens of morbid parts from the French, and even from himself, since the energetic depletive practice of the English, in active inflammations, leaves us quite destitute in that department, compared with our Continental brethren. We have, times out of number, proved the truth of this assertion, by faithful details of the nerveless practice and minute dissections of the most celebrated French physicians. It is certainly too much to be taunted with destroying the viscera of our patients by active depletion—for if we do run into excess sometimes in this way (which we do not deny) the effects would be any thing but those frightful disorganizations pointed out by our author.

of the public, who are naturally humoral pathologists, and who are never better pleased than when they have evacuated a large quantity of yellow, green, black, and especially of fetid stuff. By such a lustration they believe themselves delivered of a multitude of poisons which could not fail to corrupt the whole mass of humours. They treasure up in their memories this fortunate disgorgement, and, after a short time, imagining that the enemy must have again collected his forces, they hail with pleasure another dose of calomel and black draught from their dear doctor. The comfortable feelings and increase of appetite which they experience soon after the process of purgation, confirm them in the necessity of the measure, and the propriety of frequently repeating it. Why, indeed, should they not cherish a remedy which gives such sudden relief, and which procures for them, the very next day, the power of indulging in the luxuries of the table? The remedy is, therefore, had recourse to, twice or thrice a week;—and if, after a time, it is found that the appetite flags, then the dear doctor has in reserve bitters, tonics, and stimulants, which again create an artificial appetite—flatter their sensualities—but pave the way for a terrible retribution. For my own part, I think the English physicians should remember that they are, by this indulgence, fixing a permanent irritation in the digestive organs of their patients; and that they should resist the propensity to purgation, and consider what they are about."—*p.* 254, *vol.* i. *Examen.*

Although M. Broussais' fears respecting the effects of purgation, particularly in fevers, are greatly exaggerated, in consequence of the peculiar ideas which he entertains as to their seat in the mucous membrane of the stomach and bowels, yet there is much truth in the general principle maintained in the above passage. We are thoroughly convinced that, in a great proportion of cases, the secretions from the bowels will be slimy, and many-coloured, and bilious, and watery, and fetid—and, in short, *disordered*, so long as we continue the use of drastic purgatives, especially in chronic diseases. It is impossible that the motions can become healthy—that is to say, natural—formed—solid, under purgation; and, therefore, the advocates of this practice, should rest from their labours occasionally, and let the bowels remain in peace for a few days to see the effect. We have, times out of number, been shewn the vile secretions which were daily passing off under calomel, scammony, and the black draught, and which were carefully ranged in half a dozen or more pot-de-chambres, every morning, each according to seniority, with the right of primogeniture most carefully guarded by the nurse. We have been asked, "can we dare to interrupt the process of purgation while things are in this condition?" We have, by much persuasion, procured an armistice of three days, at the end of which, to the astonishment of patient and practitioner, the nurse would bring to light—a

"mortal coil," on which Mr. Abernethy might feast his optics and olfactories for an hour. But we have seen other terminations of the affair; we have known the most serious diseases to be produced in the rectum and lower part of the colon by this hyper-purgative system. We have known more than one or two instances of fatal exhaustion produced by "following up," as it is called, the purgative plan, in convalescents who happened to shew some disordered secretions in consequence of improper, or too much, food. One dose of purgative medicine would, under such circumstances, be beneficial; but when it is *pursued* with the vain hope of seeing healthy motions come forward, most injurious consequences may result. In acute diseases, we well know the advantage of keeping up a brisk action in the whole line of the intestinal canal, by means of which, the whole vascular system is emptied or relieved; but this is a very different case from chronic and apyrectic affections, in which hypercatharsis, daily repeated, may and does produce serious mischief. The great object in almost all chronic disorders is to procure *one* copious, fæculent, and soft, *not liquid* motion, daily. This is quite enough—and, at this rate, the process may be carried on a long time. But beware, in chronic diseases, how you *repeat* your colocynth at night, and black dose in the morning, for days or weeks! And now to return to our critical author.

The English have the most confused and contradictory notions respecting typhus and other fevers. They cannot determine as to the seat or nature of fever. Some bleed—others give bark and stimulants—but all are agreed in one measure—*daily purgation*. The editor of this journal next comes in for a slight chastisement. He had strongly commended the ingenious views which were broached, a few years ago, in a little work written by M. Vialle, a disciple of Broussais—and for this he is treated as—" un homme de mérite."—p. 257. But, for employing calomel in fevers, he is condemned as a disciple of Brown or Rassori, and asserted to be *unacquainted with the writings of Broussais!* " Il resulte de ces reflexions que notre confière d'Angleterre, bien qu'avec l'intention de se montrer impartial, a cedé a l'influence de la doctrine Empirico-Brownienne de son pays, et que cette influence l'a exposé, malgrè ses bonnes intentions, aux reproches d'inconsequence et de légèreté."—258. To this we make no reply; but, to the charge of not being acquainted with the writings of M. Broussais, we have only to say, that, before this accusation was made, we laid before the English profession minute and comprehensive analyses of all the works which M. Broussais had then published.

After this, we might very easily turn the last line of the above passage on the author, and, with justice, expose him, "aux reproches d'inconsequence et de légèreté." But we excuse M. Broussais, because he is unacquainted with our language or literature.

Dr. Brennan and others, our author observes, give oil of turpentine in puerperal fever. In a few instances, Nature is able to combat both the disease and the doctor, but in the majority of cases, this frightful stimulation (stimulation effroyable) augments prodigiously the inflammation, and causes death in the midst of the most horrible torments—or, if not sudden death, a slow disorganization with marasmus, which is worse.

It is true, the College of Physicians of London do not sanction this "therapœia perturbatrix;" but then they have substituted a medley of antiphlogistics and excitants—that is, an irrational treatment. The English still maintain the existence of idiopathic fevers—they know nothing of the real nature of diseases of tropical climates, in which they still bleed and purge, instead of applying leeches to the epigastrium, and keeping their patients on lemonade. I am not quite sure whether or not the English know that there is such a disease as chronic peritonitis; "but the London Medical Repository cites, as a very curious case, an adhesion between the several viscera of the abdomen, and does not give the disease a name."—267. Dr. Scudamore, however, has marked an æra in medicine, by his work on Gout—" un ouvrage, *ex professo*, qui vient de faire faire à la médecine un nouveau pas."—268. The reader may be curious to know why our learned countryman has met with so mighty an elevation all at once above the pigmies of this nation. It is this:—1st, His work is translated into French—hence Broussais was enabled to read an English book—secondly (and principally) because "the fundamental idea of this author is to attribute gout to the progressive development of an *irritation in the digestive organs*." If Dr. Eady or Dr. Lynch had broached such a doctrine in this country, they would have been placed over the heads of a Baillie, a Parry, or even a Hunter! They would have been represented as making "un nouveau pas à la médecine." And after all, it is only *in theory* that Dr. Scudamore has made the "nouveau pas"—he has left the *practice* just where he found it. "La partie therapeutique ne s'est point ameliorée entre les mains de l'auteur Anglais."—275.

The English, says M. Broussais, have profited so little by the lessons of their own Sydenham or Cullen that they attempt to cure *acute rheumatism* by mechanical compression, which generally drives the irritation in upon the vital viscera!—Oh, Dr. Balfour! what ridicule have your bandaging and your pummelling brought upon the country of Sydenham and Cullen!

The English have, however, extended the boundaries of medicine in some respects. They have *invented* several new diseases. Among these is "*delirium tremens*," an erithism of the brain produced by irritation of the stomach. They pretend to cure it by opium and other stimulants, and the enemies of the new doctrine have cited this (false) fact against us; but this is all stuff. The English did not cure delirium tremens by any such means—and if they did, they *ought not* to do it—for it is against all the rules of science.

The English often think they discover new diseases and phenomena, which are well known to their neighbours. Thus Dr. Philip has published a paper in the Medico-chirurgical Transactions, on a disease which he calls *dyspeptic phthisis*—a disease described by Hippocrates, and well known ever since the days of the father of medicine. And after all, Hippocrates and Philip, and the various intermediate authors are wrong; for the disease does not begin in the *liver*, as they pretend; but is seated in the mucous membrane of the stomach and bowels.

We shall not follow M. Broussais any farther in his long review of the state of medicine in England—in which there is much of misconception, rather than of misrepresentation, all owing to his ignorance of our language, and, consequently, imperfect acquaintance with our best authors. We, therefore, absolve him from all blame, except that of setting himself up as the critic of a whole nation, without any adequate knowledge of his subject.

In thus giving a very slight exposé of the opinions and practice of M. Broussais, we beg distinctly to acknowledge, that the *principia*, or, as he terms them, "propositions of medicine," to the number of 468, occupying nearly 150 pages of his work, contain a vast fund of sound and enlightened views, which are well deserving of attentive meditation. They highly merit a translation; and it is our intention to dedicate an article to the pathological and therapeutical divisions, in the next, or an early number of this journal.

